# Spatiotemporal effects of urban sprawl on habitat quality in the Pearl River Delta from 1990 to 2018

**DOI:** 10.1038/s41598-021-92916-3

**Published:** 2021-07-07

**Authors:** Jiansheng Wu, Xuechen Li, Yuhang Luo, Danni Zhang

**Affiliations:** 1 Key Laboratory for Urban Habitat Environmental Science and Technology, School of Urban Planning and Design, Peking University, Shenzhen, 518055 People’s Republic of China; 2 Key Laboratory for Earth Surface Processes, Ministry of Education, College of Urban and Environmental Sciences, Peking University, Beijing, 100871 People’s Republic of China

**Keywords:** Urban ecology, Ecology, Biodiversity

## Abstract

Since the implementation of the Chinese economic reforms. The habitat quality of coastal has gradually deteriorated with economic development, but the concept of "ecological construction" has slowed the negative trend. For quantitative analysis of the correlation between the Pearl River Delta urban expansion and changes in habitat quality under the influence of the policy, we first analyzed the habitat quality change based on the InVEST model and then measured the impact of construction land expansion on the habitat quality through habitat quality change index (HQCI) and contribution index (CI) indicators. Finally, the correlation between urbanization level and habitat quality was evaluated using geographically weighted regression (GWR) and the Self-organizing feature mapping neural network (SOFM). The results indicated that: (1) during the study period from 2000 to 2020, habitat quality declined due to urban sprawl, indicating a deterioration of ecological structure and function, and the decrease was most significant from 2000 to 2010. (2) The urbanization index had a negative effect on the habitat quality, but the negative effect have improved after 2000, reflecting the positive effect of policies such as "ecological civilization construction" (3) The implementation degree of ecological civilization varies greatly among cities in the study area: Shenzhen, Dongguan, Foshan, and Zhongshan have the best level of green development. These results reflect the positive role of policies in the prevention of damage to habitat quality caused by economic development and provide a reference for the formulation of sustainable urban development policies with spatial differences.

## Introduction

Habitat quality refers to the ability of an ecosystem to provide suitable living conditions to sustain a species, which can reflect the level of biodiversity and ecological services to a certain extent^1,2^. Urbanization is the main driving factor that puts tremendous pressure on biodiversity conservation^3^. Since the implementation of the reform and opening-up policy, China’s urbanization rate has increased rapidly, from 17.9% in 1978 to 60.6% in 2019, and is expected to reach 65.5% by 2025. The rapid urbanization process and high-intensity human activities have converted a large number of natural habitats into construction land^4^, which has severely damaged the quality of the habitats, leading to the loss of biodiversity and causing irreversible damage to the health of the ecosystem and human well-being^5–7^. Reducing the impact of urban expansion on habitats to achieve a sustainable urban ecosystem in which humans and nature can coexist harmoniously has become a key goal of the government and urban planners^8,9^.

At present, studies on habitat quality can be divided into two categories: (1) species or regional habitat quality assessment. Most studies involving habit quality assessment use mainly two method types; One is an indirect method that reveals changes in habitat quality by measuring the variables of specific species and their populations in different habitats, mainly through collecting habitat quality parameters data through field measurements^10,11^. For example, the maximum entropy model (MaxEnt)^12^ and bioclimatic data^13^ are used to generate species distribution models. The required parameters of such models can be adjusted according to the research purpose, and they have strong pertinence, accuracy and reliability, but they consume significant labor and material resources, so they are not suitable for long-term research in a large range of areas. Another approach is to directly measure the attributes of the habitat. The common approaches are expert-based models and ecological process models. The expert-based model can reflect the ecological status of the study area or the resources required by a specific population^14^. However, the method is highly dependent on the expert knowledge when determining the indicators. Ecological process models emphasize the threat of human activities to habitat quality. Common examples include the Integrated Assessment Model for Tradeoffs of Environmental Services and Habitat Quality (INVEST-HQ)^15^ and the Global Biodiversity Model^16^. These models not only consider the ecological process, but also have a complete habitat quality evaluation system, which can reduce the randomness of evaluation index selection. (2) The second category of studies on habitat quality are those with a focus on urban ecological protection. One type of such studies is the prediction and multi-scenario simulation of habitat quality. Such studies mostly use CA-Markov and other methods to predict the spatial pattern of habitat quality in different situations, which can provide a rigorous theoretical basis for land planning and ecological restoration^17–20^. The other type is to explore the factors influencing the habitat quality, which involves studying the relationship between land-use types, landscape patterns, and habitat quality, as well as analyzing the impact of natural factors such as DEM, temperature, precipitation, and slope aspect, or human factors such as GDP and population on the habitat quality^21,22^.

Many studies have reported that urbanization is the main driving force in the degradation of habitat quality^3,23^. Land-use cover change and expansion of construction land, population agglomeration, high-intensity human activities, industrial structure transformation, and rapid economic development have led to the degradation of habitat quality, which places considerable pressure on the maintenance of biodiversity, ecosystem service functions, and ecological security^24–27^. Previous studies have simulated land change under different scenarios based on CLUE-S (Conversions of Land Use and its Effects at Small Regional Extent), and then compared the difference in habitat quality under four scenarios using InVEST model^28^. In addition, Adnan used CA (Cellular Automata) and Markov Chain models to predict future land use types and assess the corresponding socio-economic risks^29^. They have also reported that to achieve sustainable urban ecological development, the urban growth rate and expansion direction need to be controlled based on the quantity and spatial distribution of land and population. At the same time, it is necessary to promote intensive urban development, promote fixed asset investment, and technological innovation^5,30–32^. Studies on past urban expansion indicate that urban economic development poses a considerable threat to habitats on both sides of the Shenzhen River^33^. Forest remains in Seoul, South Korea, are divided into multiple isolated habitats^34^. Attention should be paid to the protection of landscape connectivity and important ecological spaces, and biodiversity conservation should be considered an important component of urban planning^35,36^. However, most existing studies focus on the correlation between urban sprawl and habitat quality^37,38^, and few take multiple time periods in a long time series as one of the research objects. Time breakpoints are rarely set to compare correlations over multiple time periods, so it is difficult to reveal the evolution of such correlations and the role of policies over different time periods. In this study, we focus on the role of policy in mitigating the negative impacts of urbanization on habitat quality.

Many models have been widely used to assess the impact of urbanization on habitat quality. Among them, the habitat quality change index (HQCI), contribution index (CI), ordinary least squares (OLS), and geographically weighted regression (GWR) models are commonly used. Some studies have measured the correlation between urbanization level and habitat quality from the perspective of correlation^39^. Indicators, such as HQCI and CI, that can quantitatively express the above relationship, can effectively reflect the average value and total amount of habitat loss caused by the growth of construction land^2^. GWR is improved based on OLS and constructs local regression equations at each research unit, which can reflect the interaction between different variables in the ecosystem from the perspective of spatial differentiation^38,40,41^. Compared with OSL, GWR can provide more spatial heterogeneity information and decision-making reference for urban green development. In addition, as an unsupervised artificial neural network model, SOFM has the characteristics of self-adaptation, self-organization, self-learning, etc., which can be applied to distinguish the differences in ecological service value, landscape pattern, land resources, and other eco-environment-related indicators in different regions^42,43^.

Since the implementation of the reform and opening-up policy in China, many special economic zones have been established, among which the urbanization level of the Pearl River Delta urban agglomeration has developed rapidly and the encroachment on ecological space has become increasingly considerable^44^. Therefore, we used the Pearl River Delta as the study area. Based on existing cases, the night light index, population density, and land urbanization rate were selected as economic development indicators^38^. Using river basins as the research unit, the coupling between urbanization and habitat quality was quantitatively evaluated using GWR and a self-organizing feature mapping neural network (SOFM). This study reveals the spatiotemporal pattern change in habitat quality and the impact of urbanization on habitat quality in the Pearl River Delta over the past 30 years. It also demonstrates that positive development of ecological conservation policies can provide theoretical support for the construction of an ecological space and sustainable development of the urban ecosystem.

## Materials and methods

### Study area

**Figure 1:**
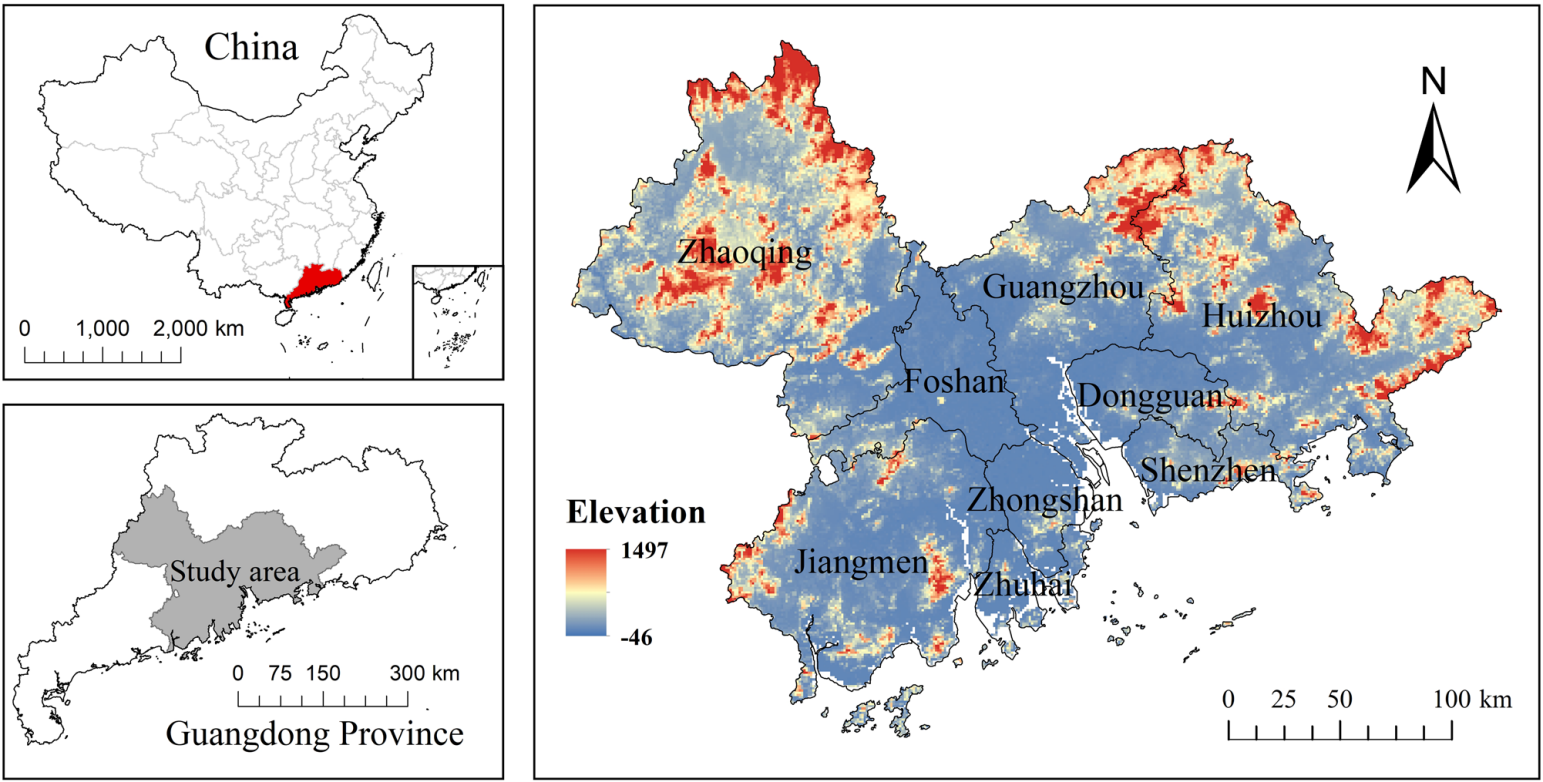
Geographical location of Pearl River Delta drawn in ArcGIS 10.6.

The Pearl River Delta (112°45′–113°50′ E, 21°31′–23°10′ N) is located in the central and southern parts of Guangdong Province, including the lower reaches of the Pearl River, adjacent to Hong Kong and Macao, and facing Southeast Asia across the sea with convenient land and sea transportation. As shown in Fig. 1, the Pearl River Delta region includes nine prefecture-level cities, namely Guangzhou, Shenzhen, Zhongshan, Zhuhai, Dongguan, Zhaoqing, Foshan, Huizhou, and Jiangmen.

### Data source

**Figure 2:**
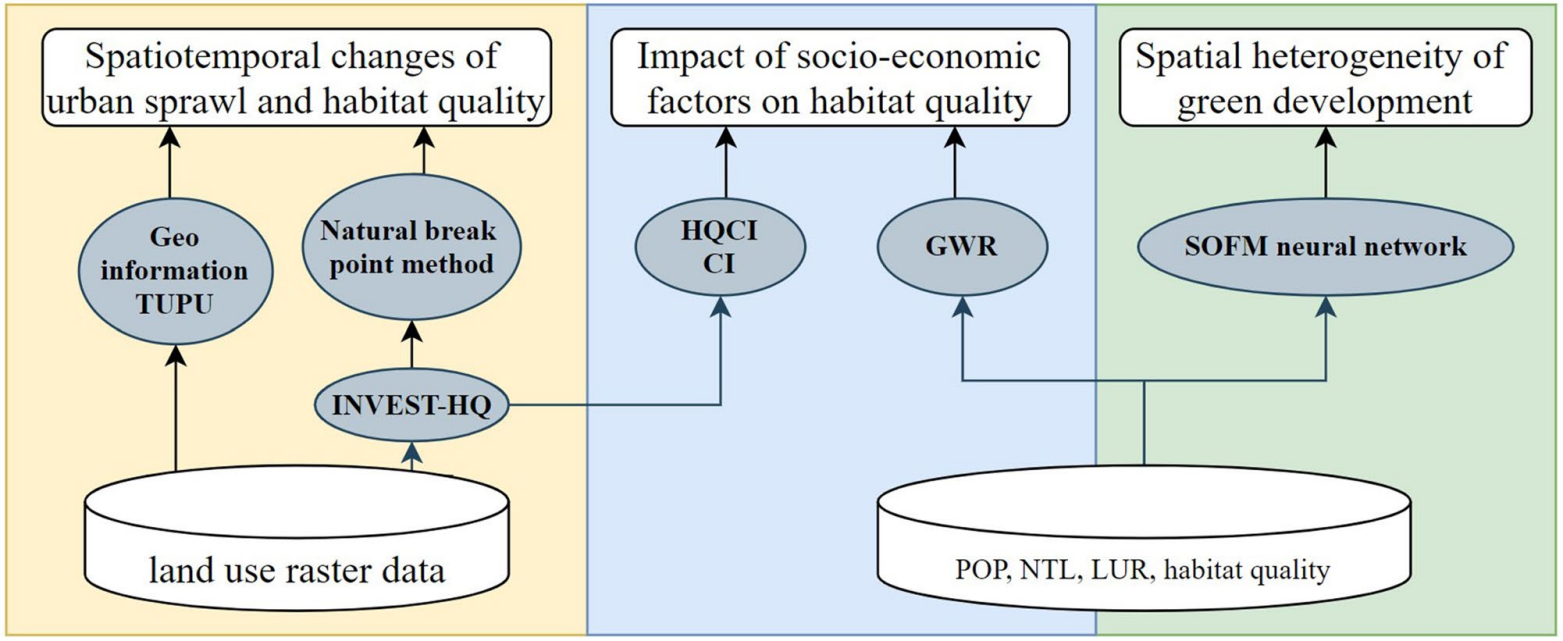
Research framework.

The research framework of this paper is shown in Fig. 2, and the data sources are as follows. Taking the basin as the research unit, the raster data of 30 m and 1 km were analyzed by zoning statistics:

1. China's land-use raster data for 1990, 2000, 2010, and 2018 were obtained from the Data Center for Resources and Environmental Sciences, Chinese Academy of Sciences (http://www.resdc.cn), with a spatial resolution of 30 m. According to land resources and their utilization attributes, the dataset divides land cover types into six first-level categories: cultivated land, woodland, grassland, water area, construction land, unused land, and land reclamation from ocean. The land urbanization rate (LUR) refers to the proportion of construction land in the whole region, which is calculated by dividing the area of construction land by the area of all land use types.
2. Raster data of population density (POP) from 1990, 2000, 2010, and 2015 were obtained from the Environment and Resources Data Cloud Platform of the Chinese Academy of Sciences, with a spatial resolution of 1 km. Owing to the stable growth of population density under normal circumstances, the population density data of 2018 were obtained by linear fitting based on POP data from 2010 and 2015.
3. Nighttime Light (NTL) raster data from 1992 to 2018 were obtained from the Nature journal data (https://doi.org/10.6084/m9.figshare.9828827.v2) with a spatial resolution of 500 m^45^ Calibration was performed to eliminate the differences in the DMSP (1992–2013) and VIIRS (2012–2018) data, generating a complete and consistent NTL dataset on a global scale.

### Land-use information TUPU

The land-use information graph is a geospatial analysis model combining attributes, processes, and spaces, which can reflect the spatial differences and temporal changes in land-use types^46^. In its function expression, let the state variables be $p\left( {p_{1} ,p_{2} ,p_{3} , \ldots ,p_{n} } \right)$, and then set *p* as a function of spatial position r and time t, as follows:

1 $$ \begin{array}{*{20}c}    {p = f\left( {r,t} \right)}  \\   \end{array} $$

where $p$ represents land-use characteristics. (1) To realize the spatial description of land attributes, when *t* is constant, the function relation of $p$ changing with $r$ is constructed. (2) The process description of land attributes can be realized, and when $r$ is constant, the function relation of $p$ changing with $t$ can be constructed. The combination of these two functions can form a conceptual model of the land-use information graph and realize a composite study of land space, process, and attributes.

### Habitat quality

#### Habitat quality evaluation

We used InVEST-HQ to evaluate the habitat quality in the Pearl River Delta region. Based on land-use types, InVEST-HQ calculated the habitat degradation degree and habitat quality index by using threat factors, the sensitivity of different habitat types to threat factors, and habitat suitability^15^. The InVEST-HQ model was co-developed by Stanford University, the Nature Conservancy, and the World Wide Fund for Nature^15^. InVEST-HQ has a low demand for data and a better spatial visualization effect, which is widely used in the field of urban ecology^47–49^. For example, The InVEST-HQ model has been used to assess dynamic changes in habitat quality in Scottish^11^, China^50,51^ and Portugal^47^. Habitat degradation and habitat quality were calculated using the following formulas:

2 $$ \begin{array}{*{20}c}    {Q_{{xj}}  = ~H_{j} \left[ {1 - \left( {\frac{{D_{{xj}}^{2} }}{{D_{{xj}}^{2}  + k^{2} )}}} \right)} \right]}  \\   \end{array} $$

3 $$ \begin{array}{*{20}c}    {D_{{xj}}  = ~\mathop \sum \limits_{{r = 1}}^{r} \mathop \sum \limits_{{y = 1}}^{y} \left( {\frac{{w_{r} }}{{\mathop \sum \nolimits_{{r = 1}}^{r} w_{r} }}} \right)r_{y} i_{{rxy}} \beta _{x} S_{{jr}} }  \\   \end{array} $$

where $Q_{{xj}}$ is the habitat quality of grid *x* in land-use type j, $H_{j}$ is the habitat suitability of land-use type j, $D_{{xj}}$ is the habitat degradation degree of grid *x* in land-use type *j*, *k* is the half-satiety sum constant, *r* is the number of threat factors, and *y* is the relative sensitivity of threat sources. $r_{y} ,w_{r}$, and $i_{{rxy}}$ are, respectively, the interference intensity and weight of the grid where the threat factor *r* is located, and the interference generated by the habitat. $\beta _{x} ,S_{{jr}}$ are the anti-disturbance ability of habitat type *x* and its relative sensitivity to various threat sources, respectively.

The value range of habitat degradation degree is [0, 1], and the larger the value, the more serious the habitat degradation. The value of habitat quality is between 0 and 1, and the higher the value, the better the habitat quality.

4 $$ \begin{array}{*{20}c}    {Linear\,attenuation:~i_{{rxy}}  = 1 - \left( {d_{{xy}} /d_{{r\,max}} } \right)}  \\   \end{array} $$

5 $$ \begin{array}{*{20}c}    {Exponential\,decay:~i_{{rxy}}  = exp\left[ { - 2.99d_{{xy}} /d_{{r{\text{~}}max}} } \right]}  \\   \end{array} $$

where $d_{{xy}}$ is the straight-line distance between grids x and y, and $d_{{r\,max}}$ is the maximum threat distance of threat factor r.

Five categories of documentation are prepared before using InVEST-HQ: LULC maps, threat factor data, threat sources, accessibility of degradation sources, habitat types and their sensitivity to each threat. Threat sources were divided into Cropland, City/town, Rural settlements, Other construction land, Unused land, and land applications. The maps of threat sources are generated in ArcGIS. For example, in the map of threat sources of cultivated land, the raster value of cultivated land is set to 1, and the raster value of other land types is set to 0. Distance between habitats and threat sources, weight of threat factors, decay type of threats factors, habitat suitability and the sensitivity of different habitat types to threat factors were derived from previous studies in similar regions^2,25,38,39,50^ and user guide manual of InVEST model^15^, as shown in Tables 1 and 2.

**Table 1:** Threat factors and related coefficients.

Threat factor	$d_{{r\,max}}$(km)	Weight $w_{r}$	Distance–decay function
Cropland	5	0.5	Exponential
City/town	9	1.0	Exponential
Rural settlements	6	0.6	Exponential
Other construction land	2	1.0	Exponential
Unused land	1	0.4	Linear
Land reclamation	2	0.3	Linear

**Table 2:** Sensitivity of habitat types to each threat factor.

Habitat type	Habitat suitability	Sensitivity					
		CUL	CL	RS	OCL	UL	DO
Cropland	0.4	0.0	0.8	0.6	0.7	0.4	0.4
Forestland	1.0	0.7	0.9	0.8	0.8	0.5	0.5
Bush forest	1.0	0.6	0.8	0.6	0.7	0.4	0.4
Sparse woodland	0.8	0.7	0.8	0.7	0.8	0.5	0.5
Other woodland	0.6	0.7	0.8	0.7	0.8	0.4	0.4
High cover grassland	0.9	0.6	0.7	0.7	0.7	0.7	0.7
Medium cover grassland	0.8	0.6	0.7	0.7	0.7	0.7	0.7
Low cover grassland	0.6	0.6	0.7	0.7	0.7	0.7	0.7
Water	0.8	0.4	0.7	0.6	0.7	0.4	0.4
Unused land	0.4	0.3	0.5	0.4	0.5	0.0	0.0
Land reclamation	0.0	0.0	0.0	0.0	0.0	0.0	0.0

CUL, cultivated land; CL, construction land; RS, rural settlement; OCL, other construction land; UL, unused land; DO, decreased ocean.

#### Habitat quality change index and contribution index

The CI was used to analyze the causes of the changes in habitat quality, and the following formula was used to qu^2,25,38,39,50^antitatively represent the contribution of land-use conversion to habitat quality change. In this study, the total value of habitat quality loss caused by land transfer in areas related to construction land expansion from 1990 to 2018 can be expressed as follows:

6 $$ \begin{array}{*{20}c}    {CI~ = ~\frac{{\mathop \sum \nolimits_{1}^{n} \left( {Q_{{ij2018}}  - Q_{{xj1990}} } \right)}}{n}}  \\   \end{array} $$

where *n* is the grid number of cultivated land transferred to construction land.

To analyze the relationship between land-use change and habitat quality, the HQCI was constructed to describe the mean value of habitat quality reduction caused by land transfer in the areas related to construction land expansion during the study period. The formula is as follows:

7 $$ \begin{array}{*{20}c}    {HQCI~ = CI_{{ij}} /S_{{ij}} }  \\   \end{array} $$

where $CI_{{ij}}$ represents the total value of habitat quality change when land-use type $i$ is converted into land-use type $j$, and $S_{{ij}}$ represents the area converted from land-use type $i$ into land-use type $j$. The positive and negative values of HQCI, respectively, represent the positive and negative impacts of land-use change on the habitat, and the higher the absolute value of HQCI, the greater the impact.

### Correlation analysis

#### Geographically weighted regression

Based on traditional OLS, GWR establishes local spatial regression and considers spatial location factors, which can effectively analyze the spatial heterogeneity of various elements at different locations^52^. The calculation formula is as follows:

$$ Y_{i}  = ~\beta _{0} \left( {\mu _{i} ,v_{i} } \right) + \sum k\beta _{k} \left( {\mu _{i} ,v_{i} } \right)X_{{ik}}  + \varepsilon _{i} $$

where $Y_{i}$ is the coupling coordination degree of the *i*th sample point, $\left( {\mu _{i} ,v_{i} } \right)$ is the spatial position coordinate of the *i*th sample point, $\beta _{k} \left( {\mu _{i} ,v_{i} } \right)$ is the value of the continuous function $\beta _{k} \left( {\mu ,v} \right)$ at $\left( {\mu _{i} ,v_{i} } \right)$, $X_{{ik}}$ is the independent variable, $\varepsilon _{i}$ is the random error term, and *k* is the number of spatial units.

To simplify the complicated urbanization process, it was divided into three aspects: economic urbanization, population urbanization, and land urbanization according to the existing research^38^. The NTL, POP, and LUR were used to represent the economic development, population scale, and land urbanization level of the city.

The research unit is a river basin, which has both natural and social attributes. It is a relatively independent and complete system, which can connect and explain the coupling phenomenon of society, economy, and nature^53^. The hydrological analysis module in ArcGIS was used to divide the research area into 374 small basins. When calculating the cumulative flow of the grid, 100,000 was used as the threshold value, and basins less than 5 km^2^ were combined with the adjacent basins.

#### Zone classification using the Self-organizing feature mapping neural network

The SOFM neural network was proposed by Kohonen, a Finnish scholar, and constructed by simulating a "lateral inhibition" phenomenon in the human cerebral cortex. It has been widely applied in classification research in geographic and land system science^42,43^. The advantages of the SOFM neural network in classifying the coupling relationship between urbanization and habitat quality are as follows : (1) it simulates human brain neurons through unsupervised learning, which is objective and reliable. (2) It maintains the data topology during self-learning, training, and simulation to obtain reasonable partition results and identify the differences between different basins. (3) For massive data, the SOFM network has a good clustering function while maintaining its characteristics and uses the weight vector of the output node to represent the original input. The SOFM neural network can compress the data while maintaining a high similarity between the compression results and the original input data^54^. We exported the data from ArcGIS, and conducted cluster analysis on the four factors of NTL, POP, LUR and habitat quality using SOFM. Finally, the analysis results are imported into ArcGIS for display.

## Results

### Spatiotemporal changes of urban sprawl and habitat quality

#### TUPU of urban expansion and construction land increase

**Figure 3:**
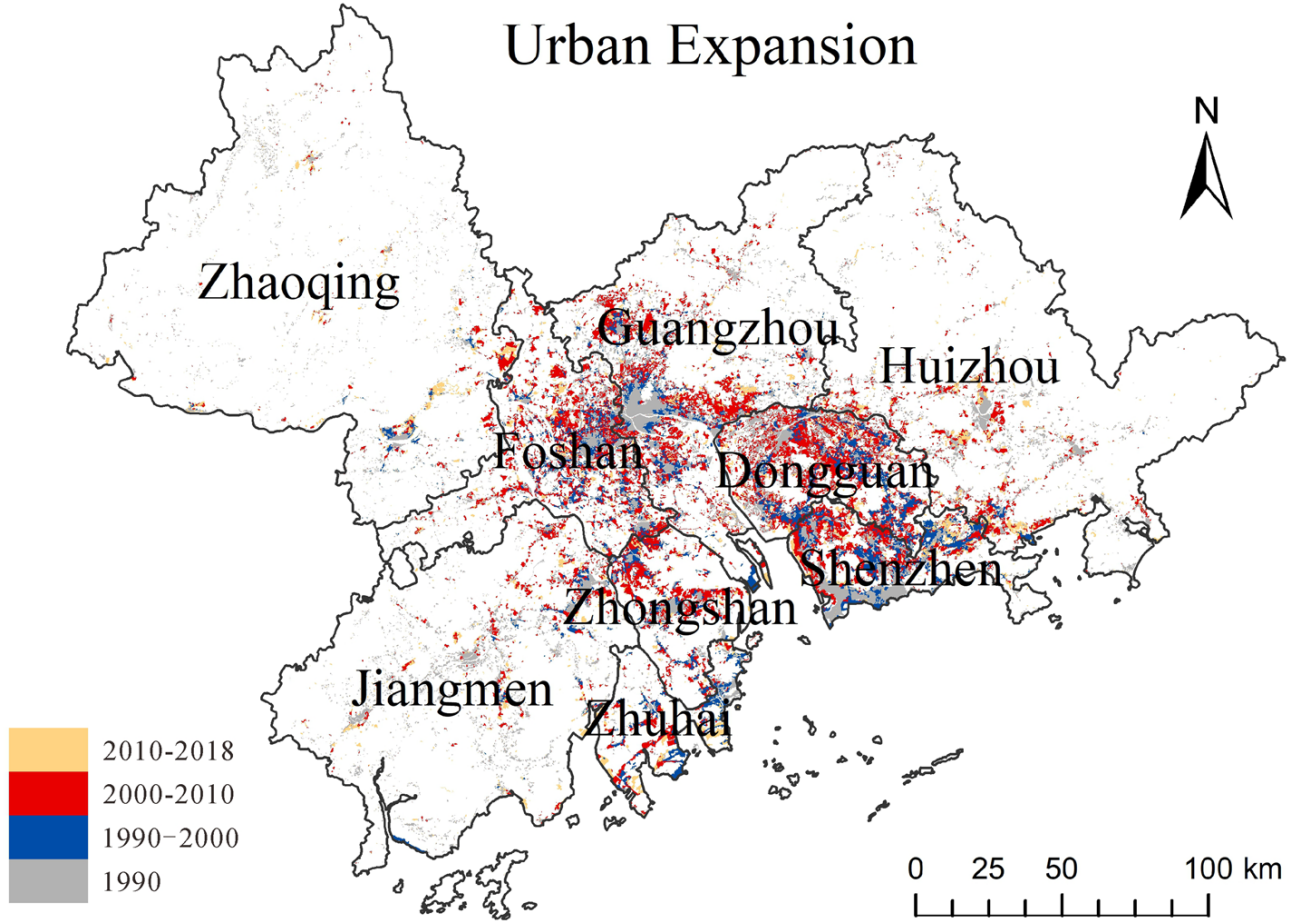
Urban Expansion map of the Pearl River Delta from 1990 to 2018.

**Figure 4:**
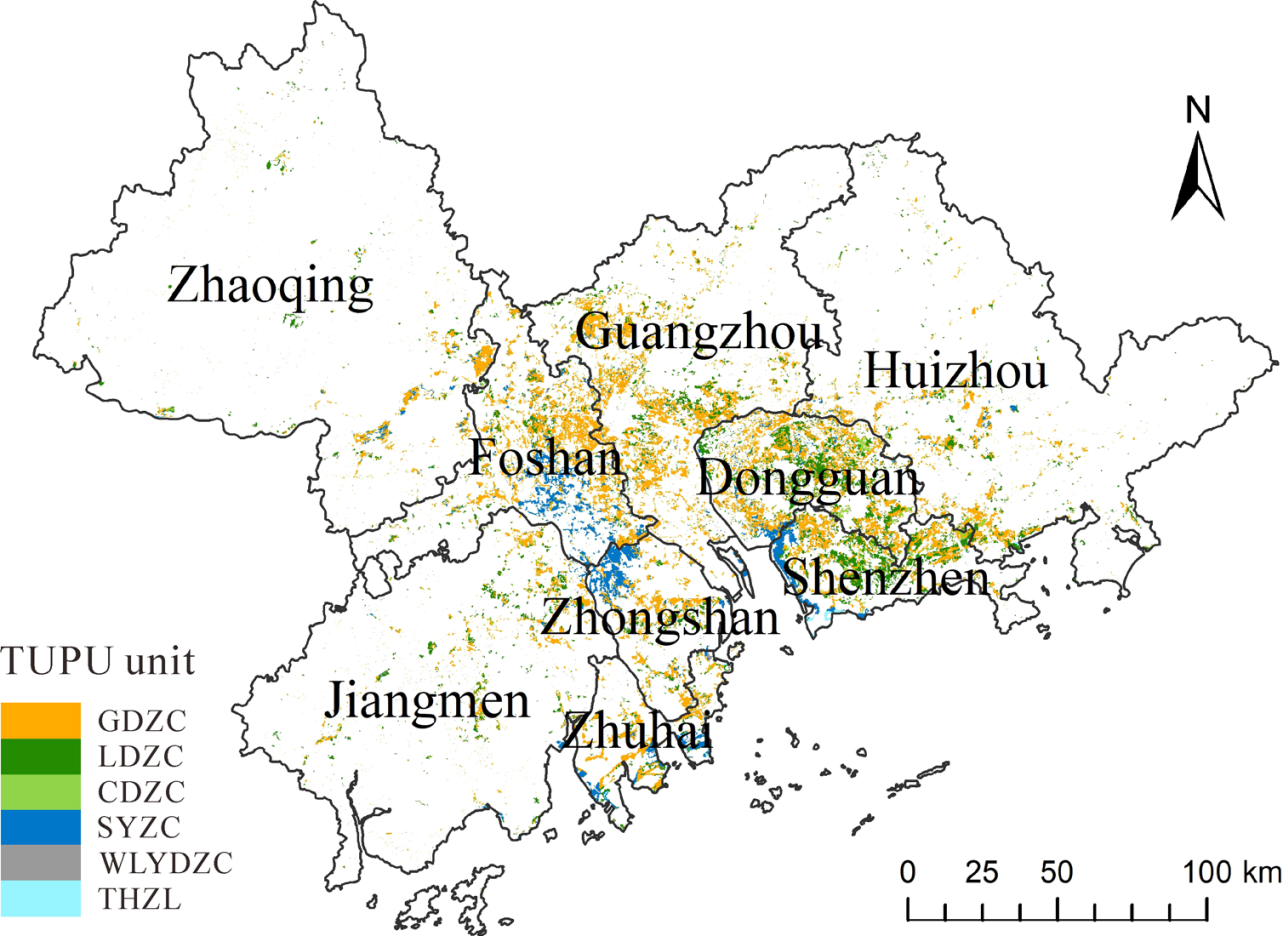
Map of construction land growth from 1990 to 2018. GDZC, decreased cultivated land; LDZC, decreased forest land; CDZC, decreased grassland; SYZC, decreased water; WLYDZC, decreased unused land; THZL, decreased ocean.

Before analyzing the spatiotemporal change of habitat quality and its degree of coupling with urbanization level, we examined the urban expansion in the Pearl River Delta region from 1990 to 2018. Figure 3 shows the growth of construction land in the Pearl River Delta region during the study period, and Fig. 4 displays the sources of construction land expansion in the region.

Figure 3 shows the spatial–temporal changes in urban expansion speed over the past 30 years. The urban expansion rate was the fastest from 2000 to 2010, and the area of construction land increased considerably more than the other two periods, and it was mainly concentrated in the central region of the Pearl River Delta, including Dongguan, Shenzhen, Foshan, Guangzhou, and Zhongshan. The area of urban expansion from 1990 to 2000 was second only to the above period, and the cities with the largest increase in construction land area during the period were also the same, but the spatial development of construction land during the period was mainly to the southeast. From 2010 to 2018, the Pearl River Delta region began to vigorously promote the reform of the ecological civilization system, and the speed of urban expansion slowed considerably. Moreover, the growth of construction land had a spatially discrete distribution. The growth area of each city was similar but considerably less than that during the other two periods.

Figure 4 shows the main sources of the expansion of construction land. The area of cultivated land was the largest among all land types transferred to construction land. The transfer of cultivated land mainly occurred in Guangzhou, Dongguan, Foshan, Shenzhen, and Zhongshan, and had a cluster distribution. The large decrease in cultivated land reflected the irreversible damage caused by the expansion of construction land to agricultural areas. The area of forestland transferred to construction land was second only to cultivated land, and it was mainly concentrated in Dongguan and Shenzhen. The large loss of water area is mainly reflected in the coastal areas of Shenzhen and Zhuhai, and the inland areas of Foshan and Zhongshan, which are important factors in habitat quality degradation. Grassland, unused land, and land reclaimed from the sea had the least area transferred to construction land, which may be because their original area was not large.

#### Spatiotemporal variation in habitat quality

**Figure 5:**
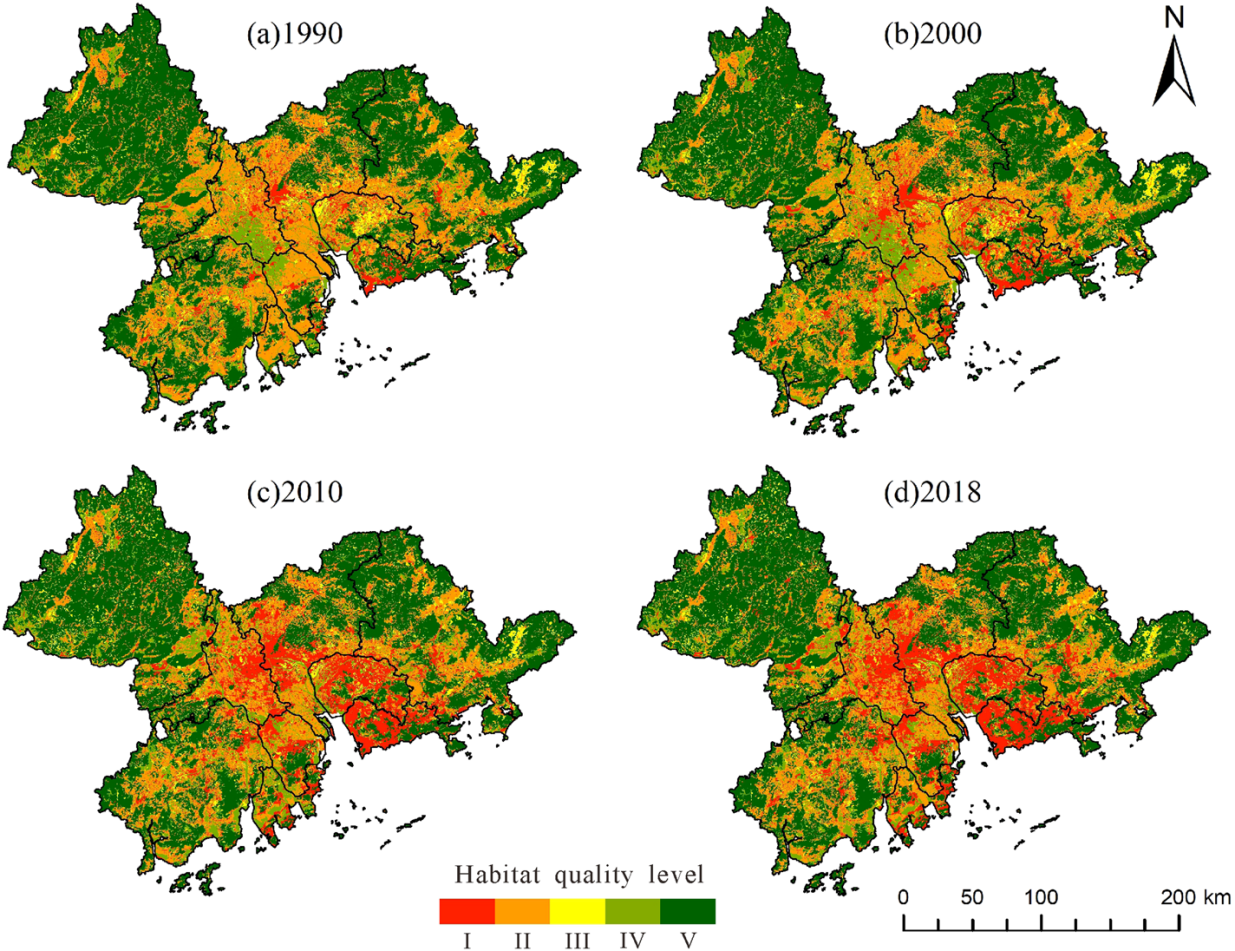
Spatial and temporal variation in habitat quality grade.

During the study period, the habitat quality in the Pearl River Delta region deteriorated with the average habitat quality index dropping from 0.7181 to 0.6672, indicating that the level of biodiversity declined and the restoration ability of the ecological environment deteriorated. To facilitate the analysis of changes in habitat quality, based on existing studies, the habitat quality index was divided into five levels and visualized as follows: low (I: 0–0.2), low (II: 0.2–0.4), medium (III: 0.4–0.6), high (IV: 0.6–0.8), and high (V: 0.8–1.0)^21^. According to Fig. 5, the degradation of habitat quality was the most obvious from 2000 to 2010, and the degradation areas were mainly concentrated in the central region of the Pearl River Delta. By 2018, the habitat quality of most areas in Foshan, Guangzhou, Dongguan, and Shenzhen had degraded to Grade I. As can be seen in Table 3, the proportion of low-grade habitat quality increased year by year, while that of high-grade habitat quality decreased year by year, and both showed sharp changes from 2000 to 2010. In 2018, the proportion of areas of low-grade habitat quality was nearly three times that of 1990, and the proportion of areas of Grade II habitat quality decreased by nearly 7%. During the study period, the occupation of ecological space by the expansion of construction land led to a considerable decrease in habitat quality.

**Table 3:** Quantitative description of changes in habitat quality levels (%).

Year	I	II	III	IV	V
1990	5.66	29.51	3.07	12.37	49.38
2000	7.96	26.79	3.25	13.23	48.76
2010	13.32	23.40	3.51	12.23	47.54
2018	14.78	22.96	3.36	11.54	47.36

**Figure 6:**
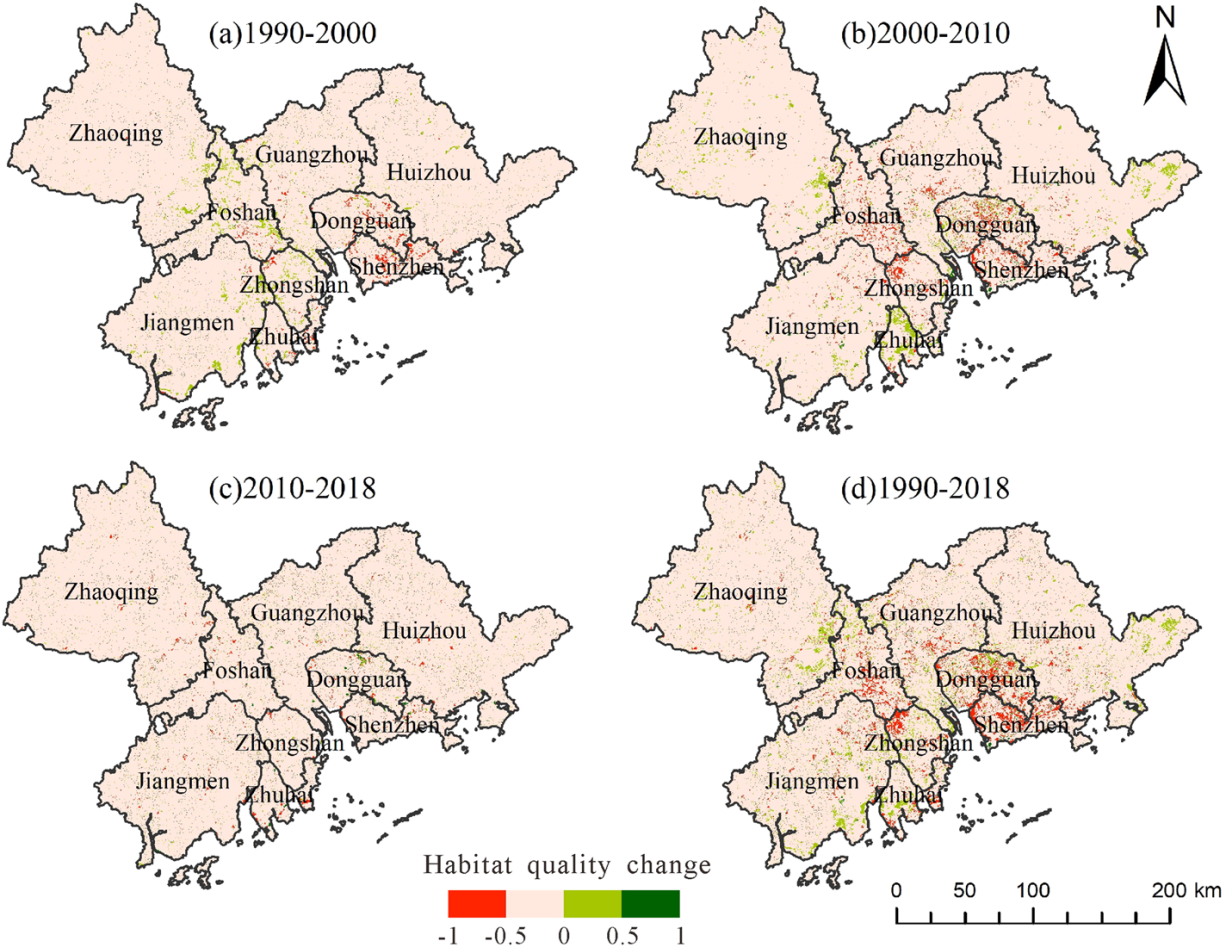
Spatial and temporal variation in habitat quality.

To reflect the spatial and temporal changes in habitat quality in the Pearl River Delta region during the study period, an interannual map of habitat quality was drawn. By comparing the three periods in Fig. 6, the area with the largest decrease in habitat quality was concentrated in Dongguan, Shenzhen, Foshan, and Zhongshan, during the period from 2000 to 2010. Second, during the period from 1990 to 2000, habitat quality decreased mainly in Dongguan and Shenzhen, while the habitat quality in some places of Foshan and Zhongshan improved. Areas with reduced habitat quality from 2010 to 2018 were scattered, and there were few areas with increased habitat quality. From observing the changes in the maps from 1990 to 2018, the areas with obvious habitat quality loss over the past three decades were mainly concentrated in Dongguan, Shenzhen, and Zhongshan, while there are many areas with increased habitat quality in Zhaoqing, Jiangmen, and Huizhou, which preliminarily indicates that these areas attach greater importance to ecological protection.

The above results can be further verified by analyzing Table 4. During the three periods, the change in habitat quality was most prominent from 2000 to 2010. The increased value of low-grade habitat quality was as high as 2893.47 km^2^, and the decrease in higher and higher quality habitat quality was more than 500 km^2^, which highlighted the negative impact of rapid economic development on ecology during the decade. In the past 30 years, the area of low-grade habitat quality increased by 4911.07 km^2^, while the sum of the area reduced to IV and V habitat quality was approximately 1500  km^2^. Substantial degradation in habitat quality has become an urgent concern. These results are consistent with the situation of urban sprawl discussed in “TUPU of urban expansion and construction land increase” section, which can preliminarily infer the correlation between urban sprawl and habitat quality.

**Table 4:** Changes in habitat quality level measured by area (km^2^).

Year	I	II	III	IV	V
1990–2000	1231.45	− 1467.49	96.70	457.77	− 344.96
2000–2010	2893.47	− 1794.23	141.36	− 521.39	− 598.84
2010–2018	786.16	− 235.64	− 78.49	− 372.51	− 91.90
1990–2018	4911.07	− 3497.36	159.57	− 436.12	− 1035.70

HQCI and CI indices were used to quantitatively analyze the impact on habitat quality of different land-use types transferred to construction land. The results are shown in Table 5. According to the HQCI, all land conversions to construction land will lead to habitat quality degradation. The HQCI was negative, and the absolute value was greater than 0.10, and the effect of grassland transfer on habitat quality was the most obvious, with an HQCI value of − 0.30. It can be observed from the CI value that the conversion of cultivated land leads to the degradation of habitat quality the most, with a CI value of − 386.02, followed by woodland and water areas. The reason why the HQCI value of these land transfers is smaller but the CI value is larger is that they cover a larger area. However, grassland conversion had the greatest impact on habitat quality per unit area, but the total loss to habitat quality was not obvious because of the small area of grassland transfer.

**Table 5:** Impact on habitat quality of land types transferred to construction land.

Transfer land type	HQCI	Transferred area	CI
Cropland	− 0.12	3090.40	− 386.02
Woodland	− 0.18	1233.74	− 216.24
Grassland	− 0.30	123.71	− 36.87
Water	− 0.18	690.39	− 121.87
Unused land	− 0.28	8.67	− 2.44
Reclaiming land from the sea	− 0.21	22.01	− 4.70

### Impact of socioeconomic factors on habitat quality

The changes in urban expansion and habitat quality reflect the important influence of human activities on the ecological space. In this study, habitat quality was taken as the dependent variable, and NTL, POP, and LUR were selected as independent variables by referring to existing studies^21,38^. The OLS and GWR were used for the analysis, and it was found that the explanatory power of the GWR model at four time points was superior to that of the OLS model, and the Sigma and AICC values of the former were lower. Therefore, the GWR model was selected to obtain a better fitting effect and higher accuracy.

**Figure 7:**
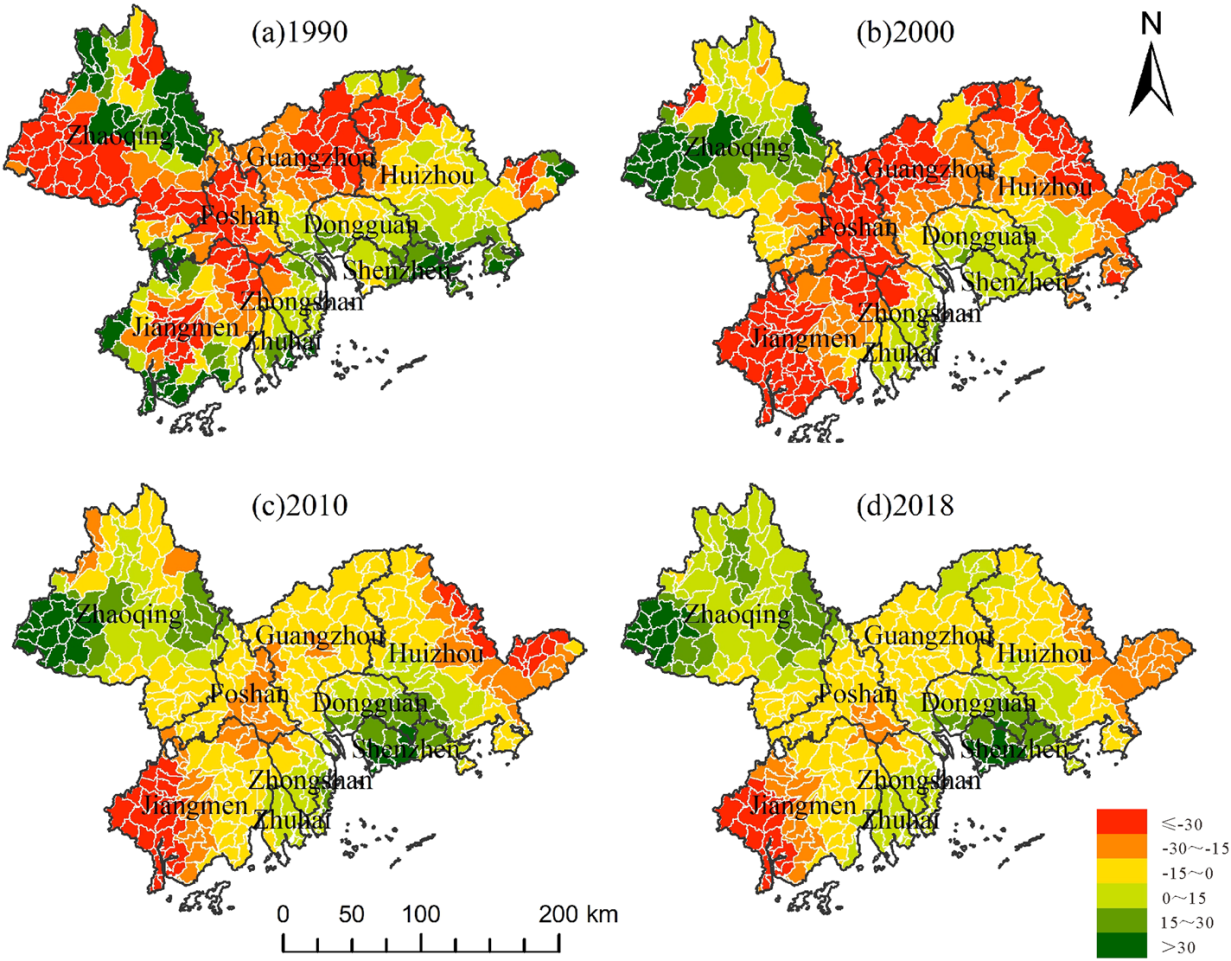
Spatial pattern of correlation coefficient between NTL and habitat quality.

**Figure 8:**
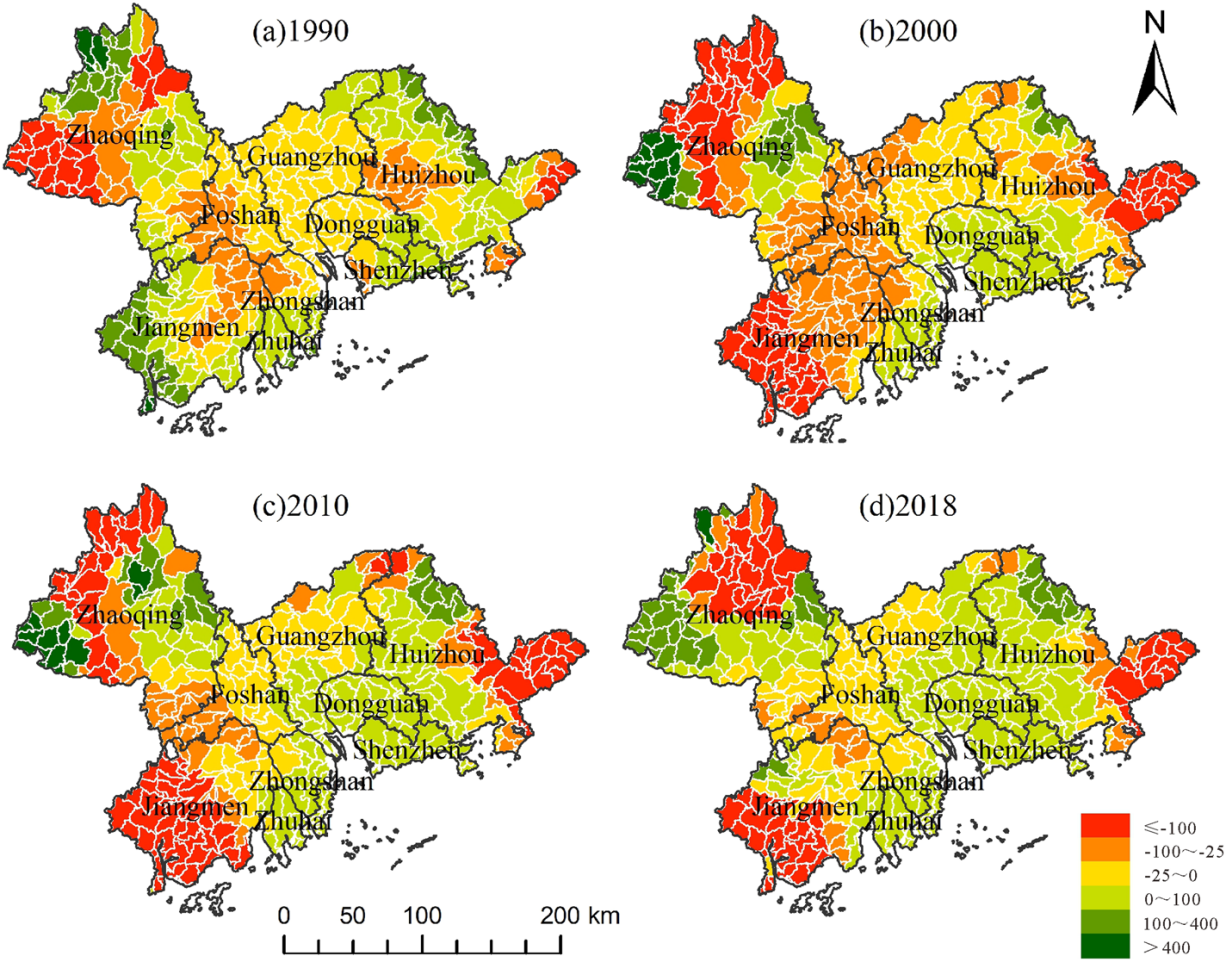
Spatial pattern of correlation coefficient between POP and habitat quality.

**Figure 9:**
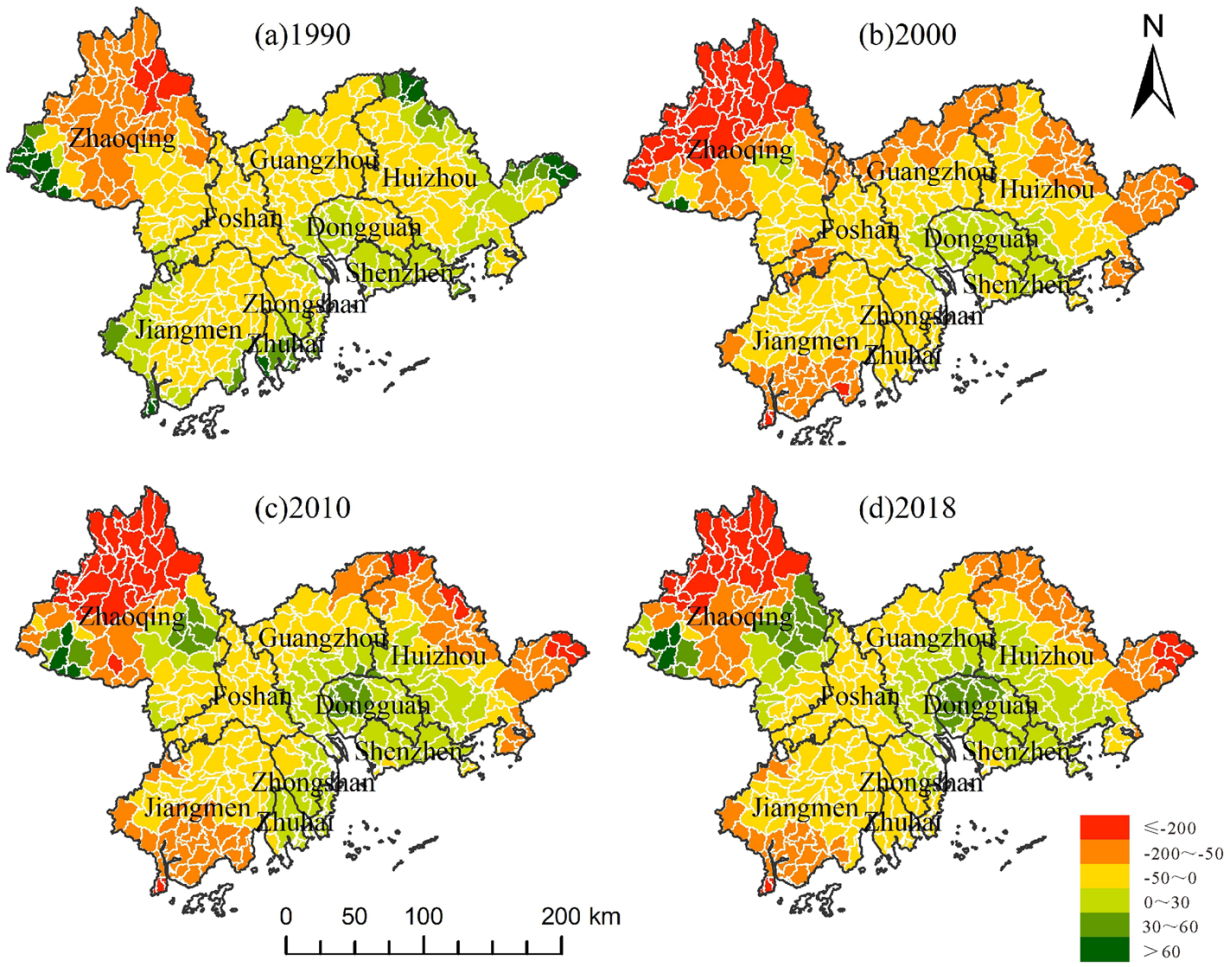
Spatial pattern of correlation coefficient between LUR and habitat quality.

During the study period, there was a negative correlation between habitat quality and the NTL, POP and LUR in most areas. With the passage of time, the negative effect first intensified and then gradually improved (Figs. 7, 8, 9). From 1990 to 2010, these three urbanization factors were negatively correlated with habitat quality in more than half of the basins, while in 2018, such a negative correlation existed in less than half of the basins. Figure 7 shows the changes in the relationship between the NTL, which represents human activities, economic development, and habitat quality. The correlation between the two time points in 1990 and 2000 indicates that the negative impact of urban sprawl on habitat quality tends to worsen during the decade. Moreover, the range of the negative impact shifted to the southeast and occupied many basins where there was a positive correlation between the two, while the range of the positive effect decreased sharply and was mainly distributed in the northwest. However, the negative impact of urbanization on habitat quality improved considerably in 2010, and the negative correlation between urbanization and habitat quality was weakened, while a positive correlation was strengthened, and this trend continued until 2018. The ratio of construction land area to total area is the LUR, which is similar to the effect of POP on habitat quality and NTL, but the expansion direction is slightly different; that is, the range of negative influence is transferred to the outer PRD, and the positive influence is strengthened in the southeast. In addition, Shenzhen, Dongguan, Zhongshan, and Zhuhai had the best ecological progress.

According to the above analysis, we can see a correlation between urbanization and habitat quality in the Pearl River Delta region. From 1990 to 2000, the scope of negative influence increased, and the degree of positive influence decreased. The main reason is that the Pearl River Delta opened as a "coastal economic development zone" in 1985. The government has vigorously developed its economy and promoted trade development. The high intensity of economic activities has seriously degraded habitat quality. From 2000 to 2010, the negative impact began to weaken, and the number of basins with a positive correlation between urbanization factors and habitat quality increased. The main reason is that the government has begun implementing the policy of "building an ecological civilization, basically forming the industrial structure, growth mode, and consumption mode that save energy and resources and protect the ecological environment" proposed in the 17th National Congress of the Communist Party of China, and has maintained a good balance between high-speed urbanization and ecological resource protection. From 2010 to 2018, it continued the improving trend of the previous decade, which was closely related to the release of the *Implementation Plan of the National Ecological Civilization Construction Demonstration Zone in the Pearl River Delta Urban Agglomeration.*

### Green development zoning

Based on the principle of "accelerating the establishment of a legal system and policy guidance for green production and consumption, and establishing a sound economic system for green, low-carbon and circular development" proposed in the report to the 19th CPC National Congress, we used the SOFM neural network to divide 374 river basins into four regions: green development, high-speed zone A, stable zone B, low speed zone C, and fragile zone D.

**Figure 10:**
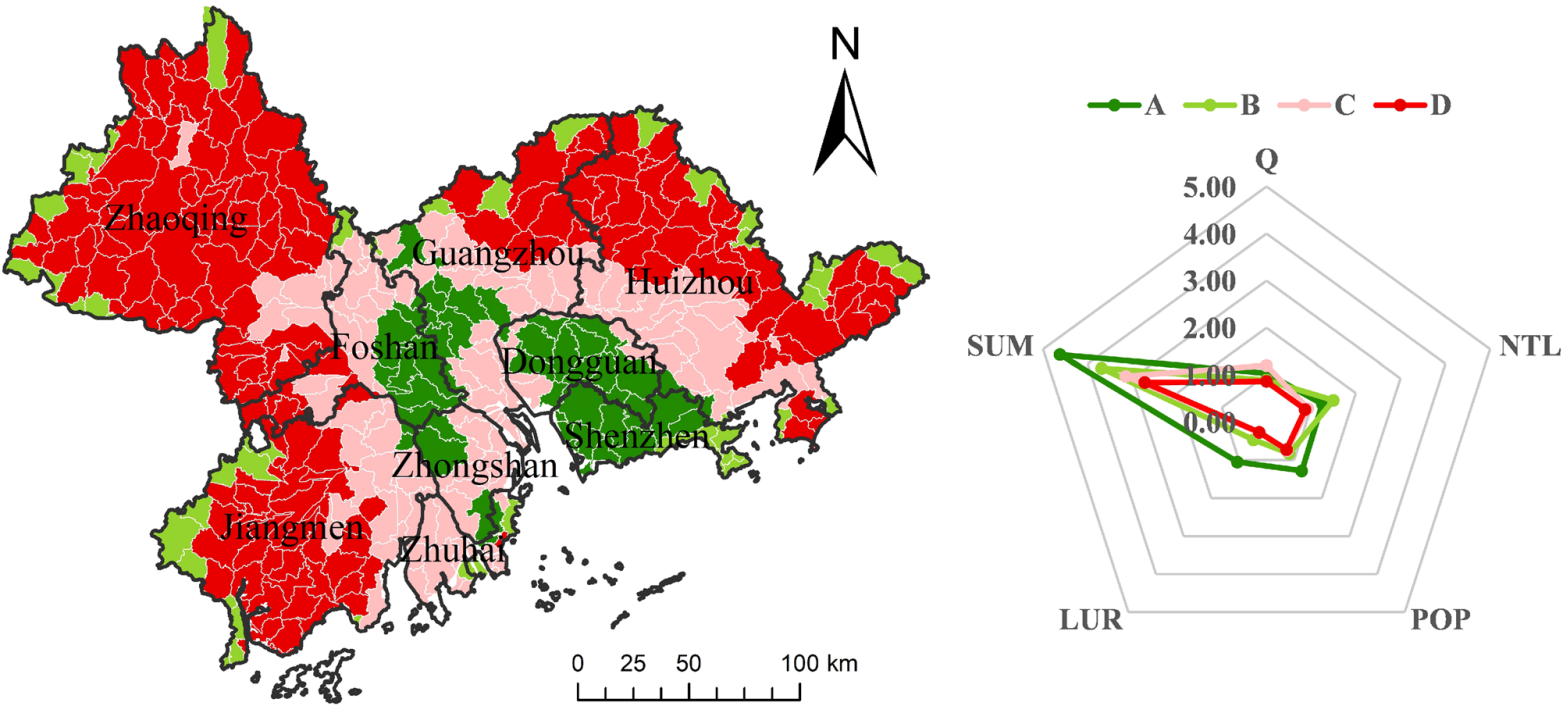
Regional distribution and characteristics of urban habitat coupling degree from 1990 to 2018. Q, habitat quality index; NTL, nighttime light; POP, proportion of population; LUR, land urbanization rate; SUM, the SUM of the average values of the four indicators (standardized) in each basin.

According to Fig. 10, cities in the Pearl River Delta vary greatly in the importance they attach to the construction of ecological civilization and green development. Shenzhen and Dongguan have the best level of green development, followed by Foshan and Zhongshan, which is consistent with the analysis results in “Correlation analysis” section. However, the urbanization level and habitat quality of most areas in Zhaoqing, Jiangmen, Huizhou, Zhuhai, and Guangzhou are relatively low, so it is necessary to explore the economic growth and social development model with efficiency, harmony, and sustainability as the goal. The gap reflects the effectiveness of "implementing the strategy of main functional zones" in the construction of ecological civilization, but it is still necessary to promote resource conservation and industrial transformation in order to achieve sustainable ecological development.

## Discussion

### Effectiveness of ecological civilization construction

Habitat loss and degradation in the process of urbanization are considered the main causes of biodiversity decline. Protecting habitat quality during urban expansion and economic development has become an urgent problem^23,30^. In recent years, when human beings exploit and utilize natural resources, they often only pay attention to their short-term economic value, but ignore the protection of habitat quality, resulting in a serious loss of ecological space and function, causing many ecological problems. These problems, in turn, affect the development of human society and the economy and have a considerable impact on human well-being and natural capital. Finding an efficient, harmonious, and sustainable economic growth and social development mode amid rapid urban development needs to be explored jointly by the government and planners.

Ecological civilization construction elevates sustainable development to the height of green development, leaving more ecological assets for future generations. Its main content can be divided into four aspects :(1) optimizing the pattern of land space development. (2) comprehensively promoting resource conservation. (3) Strengthening the protection of natural ecosystems and the environment (4) Strengthening the system to promote ecological progress. Before the twenty-first century, China attached great importance to economic development and paid little attention to the ecological environment, leading to the occupation of many ecological spaces by urban expansion. From 2000 to 2010, China began to pay attention to the common development of ecology and economy, and proposed to "build ecological civilization and basically form the industrial structure, growth mode, and consumption mode that saves energy and resources and protects the ecological environment". During the period, the degree of coordination between habitat quality and economic development in the triangular region gradually improved. For example, in the Opinions on the Implementation of Accelerating the Construction of Ecological Civilization in Guangdong Province, Guangdong Province proposed strengthening the positioning of main functions and optimizing the pattern of territorial space development. The Environmental Protection Department of Guangdong Province has issued an implementation plan for the Pearl River Delta Urban Agglomeration National Ecological Civilization Demonstration Zone to promote green development. Under the influence of these policies, the positive impact of socioeconomic factors on habitat quality has expanded and strengthened.

### Suggestions on optimizing urban ecological space

This study is helpful for regional ecological protection and urban ecological space optimization and provides a new perspective for the sustainable development of urban ecology. The results indicated that urban development-related indicators were important factors affecting the change in habitat quality, which had a negative impact on most areas in the Pearl River Delta. However, several urban and ecologically coupled areas have appeared over time. Therefore, appropriate policies can strengthen the construction of an ecological civilization and simultaneously realize social and economic urbanization at the same time. Controlling the quantity of growth and spatial distribution of land and population is conducive to realizing the common development of urban ecology. In view of the above objectives, this study suggests the following: (1) Implementing the concept of a compact city. For example, from 1990 to 2000, construction land in the Pearl River Delta region expanded radially, which accelerated habitat loss. Therefore, in the development process of new urban areas, the expansion direction and growth rate of population construction land should be controlled, and habitat segmentation should be restrained to reduce isolated ecological space. (2) Adjusting land-use patterns to adapt to urban development. For example, we should rationally plan the red line for ecological protection, optimize the spatial pattern of arable land, grassland, and construction land, and pay attention to recycling development to strengthen the protection of ecological land while maintaining reasonable economic growth. (3) Prioritizing protection of forests and grasslands in the outer suburbs first, based on the GWR analysis, although the socioeconomic development of these regions is slow, their habitat quality is strongly responsive to urbanization, and minor human disturbance can affect their ecological environment. Second, the SOFM classification results show that these areas have a low level of urbanization and habitat quality is not considerably higher than that of the Pearl River Delta core areas, so they have high development potential.

### Limitations and future research direction

Based on existing research, the following innovations were made in this study: not only quantifying the harm of urban sprawl on habitat quality, but also identifying the regional differences in three types of socioeconomic factors, and reflecting the importance of policy in urban ecological protection through comparison between different time periods. First, in order to examine the impact of policies on habitat quality in different periods, HQCI and CI index were used to quantitatively analyze the specific impact of land transfer on habitat quality dating back to the time when the Pearl River Delta was just established as a "coastal economic development zone.” Then, considering the construction of ecological civilization as the starting point, the GWR analysis results reflect the regions that pay attention to economic development but ignore the protection of ecological resources during the research period. Finally, based on the clustering results of the SOFM neural network, the differences in green development in different regions over the past 30 years were discussed. Consistent with previous studies, the results of this study also show that urban expansion and human activities have a serious negative impact on habitat quality. The difference is that some studies do not consider the spatial and temporal differences of various influencing factors and the impact of ecological-protection-related policies in different regions^25,38^. In this study, from the perspective of policy changes, the spatial heterogeneity of social and economic factors is included to provide a reference for the social and economic development process of coastal urban agglomerations and the relationship between urbanization and the ecosystem.

Although this study has effectively supplemented and expanded the sustainable development of urban ecology in the Pearl River Delta region, it still has some limitations. First, the assessment of habitat quality is a complex task. Although the InVEST -HQ model has been applied by many scholars to calculate the habitat quality index, it needs to be improved in terms of pertinence and reliability because it is based on land-use type. In the future, multi-source data will need to be considered to reflect habitat quality. Second, because of the limitation of data and considering that both population and night light are social and economic factors with stable growth in the short term, the population density in 2018 and the night light grid layer in 1990 in this study were obtained by linear fitting of the data of the most recent year, so as to maintain the consistency of data sources and research period. In future studies, more complete original data will be sought to avoid these errors. Finally, the spatial scale effect plays an important role in studies related to geography and ecology. To reflect the natural and social attributes at the same time, this study adopts the watershed as the research unit when analyzing the correlation by GWR and classifying by SOFM to solve the problem of inconsistent resolution of original data. In different analysis scales, the correlation direction, determination coefficient and relative contribution of significant factors vary with the change of analysis scales, underlining a strong scale effect. B Since the dominant drivers vary across different analysis scales, the best analysis scale should be determined according to the characteristics of the study area. We will then consider changing the size of the research unit to explore the impact of urban development on habitat quality at different spatial scales.

## Conclusions

Rapid urban expansion and high-intensity human activities have greatly affected habitat quality in the Pearl River Delta. Based on the analysis of the spatiotemporal evolution characteristics of habitat quality, the GWR model was used to explore the impact of urbanization on habitat quality, and the SOFM neural network was used to cluster each river basin into four zones according to the green development status. The habitat quality index was calculated based on the InVEST-HQ model, and urbanization indexes included NTL, POP, and LUR.

The main results are as follows: (1) The period of urban expansion was the fastest from 2000 to 2010, which coincided with the period of decreasing habitat quality, and the area of urban expansion was mainly concentrated in the center of the Pearl River Delta, including Dongguan, Shenzhen, Foshan, Guangzhou, and Zhongshan. Among the types of land transferred to construction land, arable land accounts for the largest area, causing irreversible harm to development of the agricultural level. (2) The area of low-grade habitat quality increased by 4911.07 km^2^ during the study period, and the sum of the reduction in areas of IV and V habitat quality was about 1500 km^2^. The conversion of grassland to construction land per unit area had the most obvious effect on habitat quality, while the conversion of cultivated land caused the greatest total loss of habitat quality. Considerable degradation of habitat quality has become a matter of urgent concern. (3) There were considerable negative correlations between habitat quality and NTL, POP, and LUR in most areas during the study period. Before 2000, the negative impact worsened but gradually improved from to 2000–2018, which is closely related to a large number of policies related to "ecological civilization construction" since the twenty-first century. (4) Different cities in the Pearl River Delta have great differences in the importance they attach to the construction of ecological civilization and green development. The level of green development in Shenzhen, Foshan, and Zhongshan was the highest, while the levels of urbanization and habitat quality in most areas of other cities were relatively low.
